# Biliary Adenofibroma of the Liver: Report of a Case and Review of the Literature

**DOI:** 10.4061/2010/504584

**Published:** 2010-10-28

**Authors:** Alessandra Gurrera, Rita Alaggio, Giorgia Leone, Giuseppe Aprile, Gaetano Magro

**Affiliations:** ^1^Divisione di Anatomia Patologica, Dipartimento G.F. Ingrassia, Policlinico Universitario-Vittorio Emanuele, Università di Catania, via Santa Sofia 87, 95123 Catania, Italy; ^2^Divisione di Anatomia Patologica, Universita' di Padova, 35122 Padova, Italy; ^3^Dipartimento di Chirurgia, Unità di Gastroenterologia ed Endoscopia, Università di Catania, 95123 Catania, Italy

## Abstract

We herein report the clinicopathologic features of a rare case of biliary adenofibroma (BAF) of the liver in a 79-year-old man. Grossly, tumour presented as a well-circumscribed, 5.5-cm mass with a solid and microcystic appearance. Histological examination was typical of biliary adenofibroma, showing a proliferation of variable-sized tubulocystic structures embedded in a moderately cellular fibrous stroma. Immunohistochemistry, revealing immunoreactivity of the epithelial component to cytokeratins 7 and 19, was consistent with a bile duct origin. Notably, the stromal cells had a myofibroblastic profile, showing a diffuse and strong expression of vimentin and *α*-smooth muscle actin. Differential diagnosis with Von Meyenburg complex, biliary adenoma, biliary cistadenoma, congenital biliary cystsy, and hepatic benign cystic mesothelioma is provided. The occasionally reported expression of p53 in biliary adenofibroma has suggested that this tumour could represent a premalignant lesion. The absence of both cytological atypia and p53 immunoreactivity in our case confirms that BAF is a benign tumour with an indolent clinical behaviour. However, a careful histological examination of BAF is mandatory because malignant transformation of the epithelial component has been documented in two cases.

## 1. Introduction

Benign biliary tumours are uncommon, including bile duct adenoma (also known as peribiliary gland hamartoma), biliary hamartoma (von Meyenburg complex), biliary cystadenoma, and the solitary bile cysts.Tsui et al. in 1993 described a new liver tumour entity called “biliary adenofibroma” (BAF) [1]. To the best of our knowledge, only six cases of BAF of the liver have been reported in the literature to date (Table 1) [1–6]. Two of us reported a morphologically similar tumour in equine [7]. BAF is characterized by a proliferation of tubulocystic structures variably embedded in a fibrous stroma. Etiology of BAF is still unknown, even if its immunophenotypic profile (cytokeratins 7^+^, 8^+^,  18^+^,  19^+^, D10^+^, 1F6^−^) suggests a large bile and/or interlobular duct origin [6]. Interestingly, monosomy 22, a cytogenetic alteration found in some benign mesenchymal neoplasms, has been documented in one case of BAF [5]. Although BAF is a benign tumour with a clinical indolent behavior, malignant transformation of the epithelial component [2, 3] with associated distant metastases [3] has been documented.

We herein report the clinicopathological features of a rare case of liver BAF with a benign clinical course after a 7-year follow-up period. Differential diagnostic and histogenetic considerations are discussed.

## 2. Clinical History

A 79-year-old man complained of a vague abdominal pain. Ultrasound examination and computerized tomography revealed a solid 5.5 cm mass in the right lobe of liver. Blood tests, including alpha-fetoprotein, were unremarkable. No lymphadenopathy was present. A partial liver resection was performed. No postoperative complications were noted. The patient is well with no recurrence after a 7-year follow-up period.

## 3. Materials and Methods

Surgical specimen was submitted for histological examination in neutral-buffered 10% formalin, dehydrated using standard techniques, embedded in paraffin, cut to 5 *μ*m, and stained with hematoxylin and eosin. Immunohistochemical studies were performed with the labeled streptavidin-biotin peroxidase detection system using the Ventana automated immunostainer (Ventana Medical Systems, Tucson, AZ). The antibodies tested are summarized in Table 2. Negative controls for the staining were slides stained with omission of the primary antibody.

## 4. Pathological Findings

**Figure 1:**
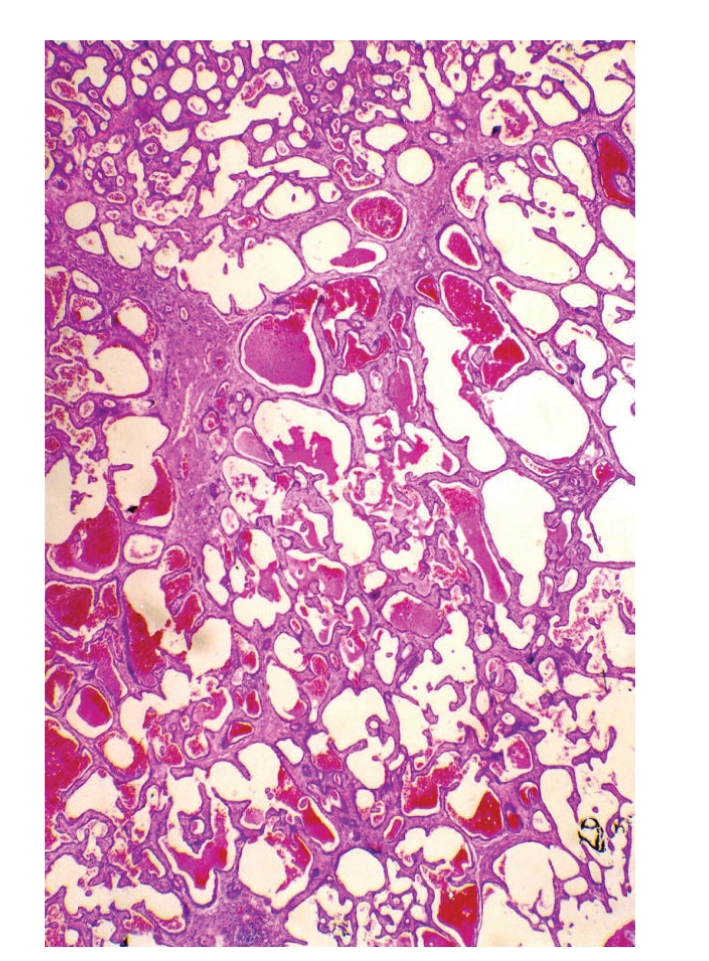
Low magnification showing a proliferation of variable-sized tubulocystic epithelial structures embedded in a fibrous stroma.

**Figure 2:**
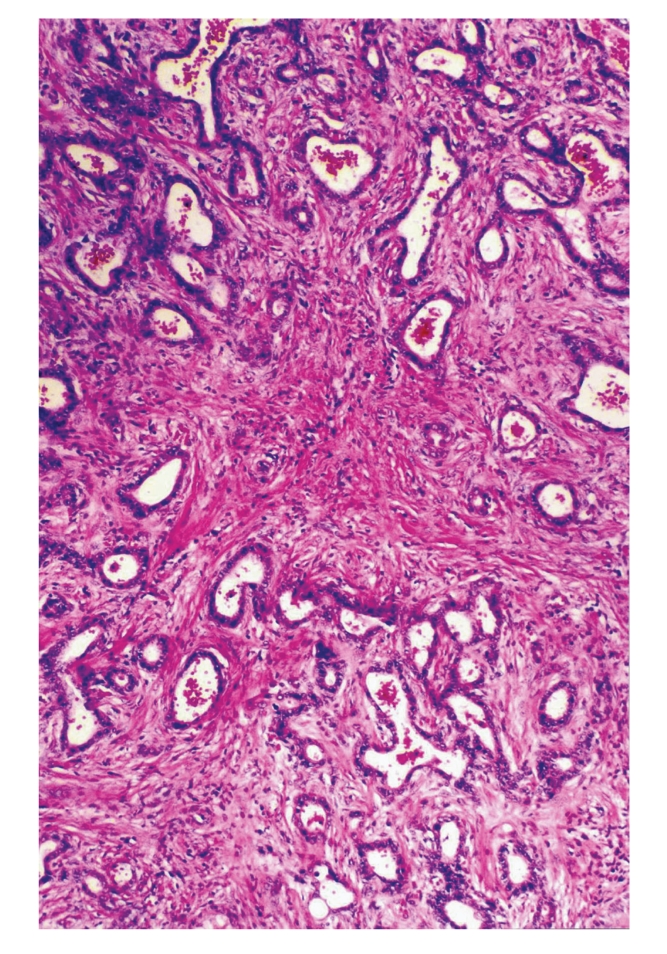
Tubules are lined by bile duct epithelium and set in a moderately fibrous stroma.

**Figure 3:**
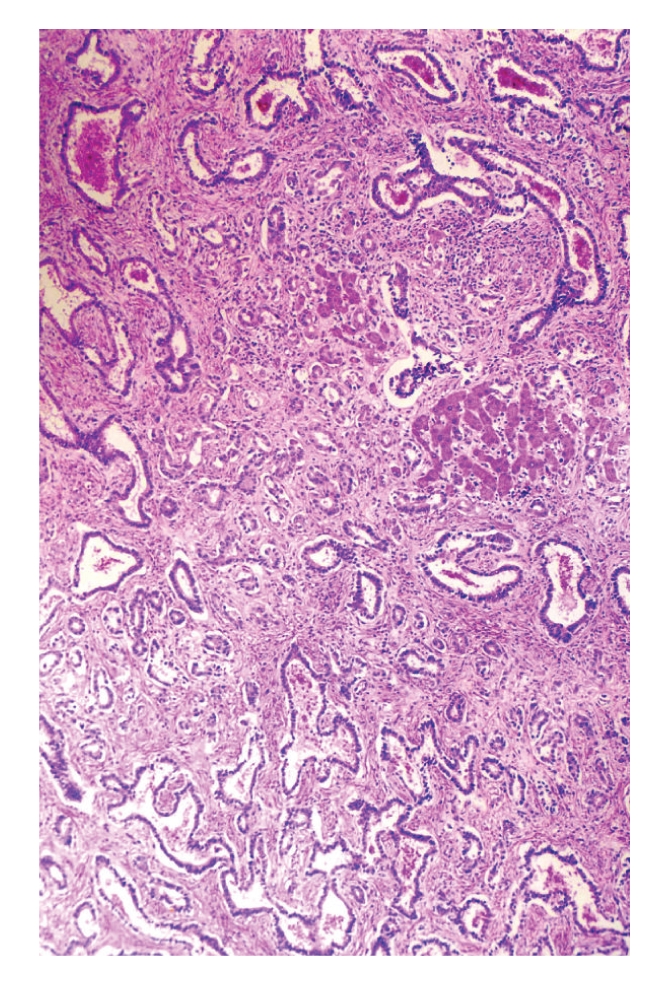
Residual hepatocytes are entrapped within tumour.

Grossly, surgical specimen consisted of liver parenchyma (14 × 10 × 4 cm) containing a 5.5-cm nodular mass merging from the liver capsule. The cut surface of the mass showed an unencapsulated, well-circumscribed, and firm lesion with a solid and microcystic appearance. Cysts ranged in size from 0.1 to 0.5 cm. Histologically, at low magnification, the tumour was composed of a proliferation of nonmucin-secreting, tubulocystic structures, embedded in a moderately cellular fibrous stroma (Figure 1). Both tubules and cysts were lined by a single layer of cuboidal to flat, bile duct-type epithelium (Figure 2). Lumens of the tubulocystic structures contained eosinophilic material and/or red blood cells, while bile was absent. Nuclei were round to oval in shape and centrally located. No goblet cells were observed. In some areas, the epithelium formed papillary projections into the lumens. Necrosis, mitoses, and nuclear pleomorphism were absent. Stromal component contained spindle-shaped fibroblast-like cells and scattered lymphocytes (Figures 1 and 2). Islands of hepatic parenchyma were seen scattered throughout the tumour (Figure 3).

**Figure 4:**
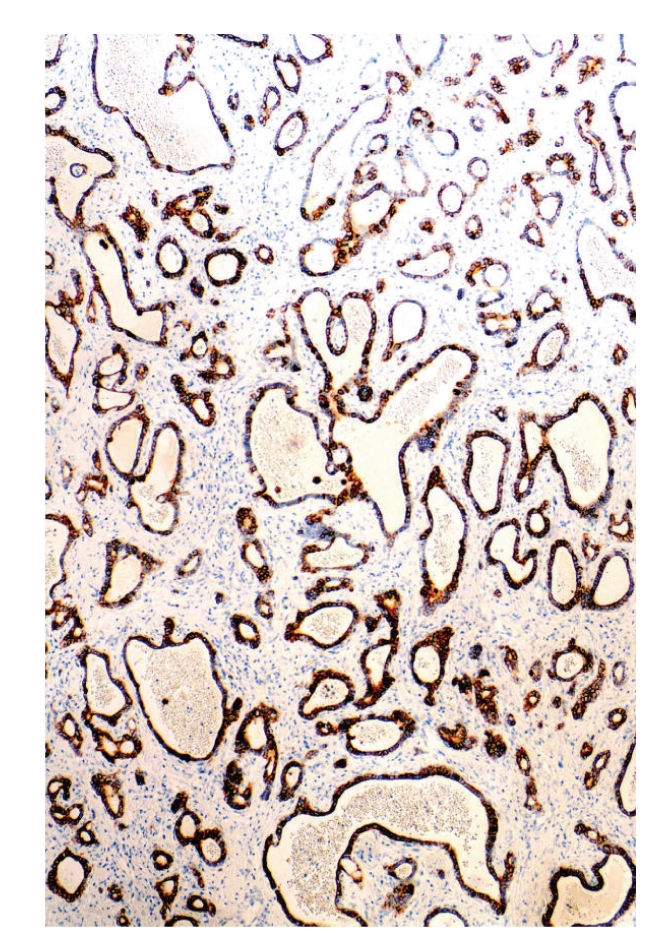
Tubules of tumour are positive for cytokeratin 19.

**Figure 5:**
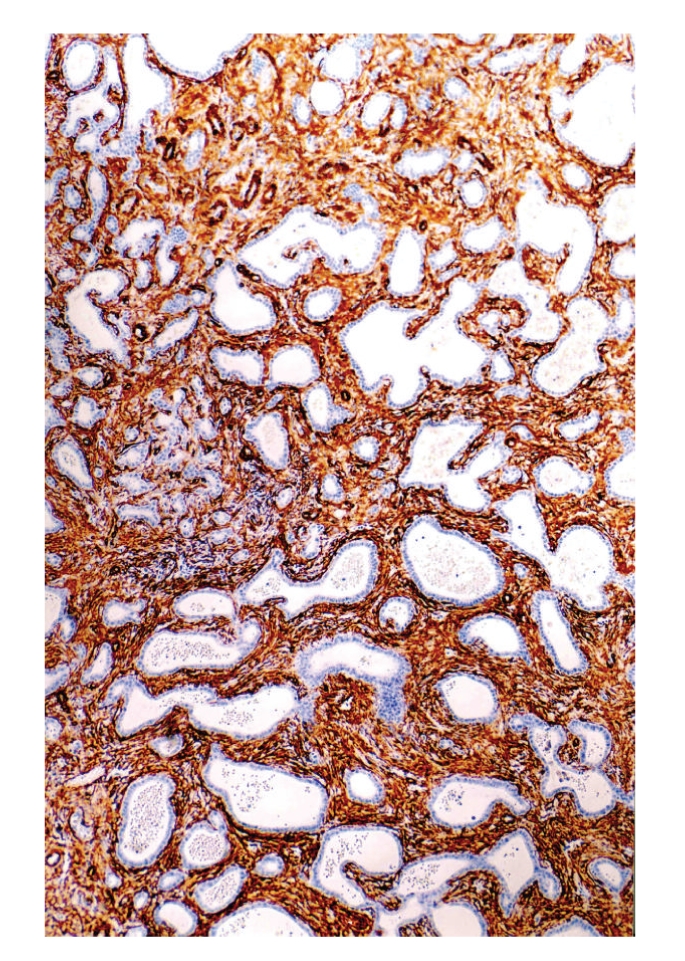
Stromal cells are strongly and diffusely stained with *α*-smooth muscle actin, revealing their myofibroblastic nature.

Immunohistochemically (Table 2), the epithelial component was stained with cytokeratins 7, 8, 18, and 19 (Figure 4) and epithelial membrane antigen (EMA). Carcinoembryonic antigen (CEA), cytokeratins 5/6, p53, calretinin, HBME-1, and beta-catenin were negative. Ki-67/index proliferation was low (1%).The stromal cells were strongly and diffusely stained with vimentin and *α*-smooth muscle actin (Figure 5) while no immunoreactivity was detected for desmin. Based on their morphological and immunohistochemical profile, these cells were regarded to be myofibroblastic in nature.

## 5. Discussion

BAF is a rare tumour of bile duct origin with only six cases reported in the literature to date [1–6] (Table 1). BAF is equally reported both in women and men ranging in age from 21 to 79 years (Table 1). Most patients complain of abdominal pain in the right hypochondrium. Imaging studies, including ultrasonography, computerized tomography and magnetic resonance imaging, usually reveal a well-circumscribed solid-cystic mass, variable in size from 5.5 to 20 cm (Table 1). Unfortunately, radiologic images, including those of our case, are nonspecific, and the diagnosis of BAF of the liver is based on histological examination [2–4, 6].

The present case has the morphological characteristic and immunohistochemical features of liver BAF. Grossly, tumour presented as an unencapsulated, well-circumscribed, 5.5 cm solid-cystic nodule. Histologically, it was composed of a tubulocystic proliferation of variable-sized bile ducts with an immunohistochemical profile (CK7^+^  , CK19^+^) suggesting a bile duct origin [1, 6]. The lumens of the tubules contained eosinophilic material and/or red blood cells but bile was absent [1, 6]. The epithelial component was embedded in a moderately cellular fibrous stroma, predominantly containing myofibroblasts as documented by a diffuse and strong immunoreactivity for *α*-smooth muscle actin. Notably, the cellular stromal component of liver BAF has been reported to be fibroblastic rather than myofibroblastic in nature, because only scattered *α*-smooth muscle actin^+^ cells have been identified [6]. As beta-catenin has been reported to be expressed in some tumours with adenofibromatous components [8], we evaluated this marker in the present case of BAF. Unfortunately, we found no immunoreactivity for this antigen either in the epithelial or stromal component.

Differential diagnosis of BAF includes von Meyenburg complex, bile duct adenoma, biliary cystadenoma, biliary cysts, and benign cystic mesothelioma. BAF generally presents as a large-sized nodule with a solid microcystic appearance, whereas von Meyenburg complex can be incidentally found either as single or multiple subcapsular nodules of small size, usually less than 5 mm in diameter [9]. This complex may represent part of the spectrum of ductal plate malformation and may be related to adult type polycystic disease [9]. Histologically, von Meyenburg complex is located within or adjacently to portal tracts and it is composed of multiple-branched bile ducts, sometimes with an angulated appearance, set in a collagenous stroma [9]. These tubular structures contain bile and/or eosinophilic material [9]. Bile duct adenoma is generally a well-demarcated, small-sized lesion with a diameter ≤1 cm, usually located directly underneath the liver capsule. Occasionally, this lesion may present as two or more nodules with a maximal diameter of 2 cm. Histologically, it is characterized by closely packed tubules with narrow lumens, lined by cuboidal bile-type epithelium, set in an edematous to dense fibrous stroma which may contain a variable amount of inflammatory cells. Unlike BAF, these tubules show more irregular outlines and less cystic configuration while stromal component is less prominent. Bile duct adenoma is not a true neoplasm but it is currently regarded as a peribiliary gland hamartoma [10] or a localized reactive ductular proliferation as result of a previous unknown injury. Unlike BAF, biliary (peribiliary) cystadenoma is a large-sized cystic tumour with multilocular appearance, in which cysts may be up to 15 cm of diameter. Two histological variants were recognized: serous and mucinous types [9]. The former consists of small cysts lined by a single layer of low cuboidal or flat epithelium with a clear cytoplasm; the latter contains cysts lined by columnar, cuboidal or flattened mucous-secreting epithelial cells with occasionally papillary projections and an ovarian-like stroma appearance, especially if the lesion occurs in women [11], with a characteristic immunoreactivity for oestrogen and progesterone receptors [12, 13]. The congenital biliary cysts (simple cysts) are lined by bile duct-type epithelium, and they may be solitary or multiple; the multiple form may be part of the polycystic disease. In our case, according to reported cases in the literature, no polycystic disease in the liver and in other organs was noted. Among the biliary cystic lesions of the liver the benign cystic mesothelioma can also be included, a rare neoplasm which may occur in the liver [14]. It is a large, partially cystic, well-encapsulated lesion, characterized by anastomosing cords of tumour cells, separated by large thick-walled vessels, closely reminiscent of a vascular neoplasm. This morphological pattern is quite different from that exhibited by the tumour herein presented. In addition, neoplastic cells of benign cystic mesothelioma are positive to calretinin, HBME-1, and cytokeratins 5/6 [14].

As far histogenesis of BAF is concerned, it is noteworthy that histological features similar to BAF and von Meyenburg complex have been obtained in animal-model of aflatoxin-induced cholangiocarcinoma [15]. These experimental findings, along with the large size, the p53 expression, and the tetraploidy status with a low S-phase being occasionally reported, strongly suggest that BAF could represent a premalignant lesion [6]. This hypothesis seems to be supported by the evidence that two cases of liver BAF underwent epithelial malignant transformation [2, 3]. However, in our case, we did not find any immunoreactivity for p53, confirming that BAF should be regarded as a benign tumour. Despite the absence of cytological atypia and p53 positivity, we emphasize that any BAF should be carefully evaluated to rule out malignancy.

## Figures and Tables

**Table 1:** Clinical features of BAFs reported in the literature.

Authors	n. cases	Age/gender	Size	Malignant component	Behaviour
Tsui et al. [1]	1	74/female	7 cm	No	No recurrence or metastasis
Parada et al. [5]	1	49/female	7.5 cm	No	No recurrence or metastasis
Haberal et al. [2]	1	21/male	20 cm	Yes (epithelial)	N.A
Garduno-Lopez et al. [4]	1	68/male	6 cm	No	N.A
Akin and Coskun [3]	1	25/male	20 cm	Yes (epithelial)	Recurrence and pulmonary metastasis after a 3-year followup
Varnholt et al. [6]	1	47/female	16 cm	No	No recurrence or metastasis after a 3-year followup
Gurrera et al. (present case)	1	79/male	5.5 cm	No	No recurrence or metastasis after a 7-year followup

Not available.

**Table 2:** Immunohistochemical findings.

Antibodies	Dilution and company	Results (Epithelial component)	Results (Stromal component)
Cytokeratin AE1/AE3	1/100 (DakoCytomation)	Positive	Negative
CK7	1/50 (Novocastra)	Positive	Negative
Cytokeratin 8/18 (CAM5.2)	1/100 (DakoCytomation)	Positive	Negative
CK19	1/50 (Novocastra)	Positive	Negative
EMA	1/200 (DakoCytomation)	Positive	Negative
CEA	prediluted (DakoCytomation)	Negative	Negative
P53	1/50 (DakoCytomation)	Negative	Negative
Ki-67	1/200 (DakoCytomation)	Positive (1%)	Positive (1%)
Vimentin	1/50 (DakoCytomation)	Negative	Positive
*α*-smooth muscle actin	1/50 (DakoCytomation)	Negative	Positive
Desmin	1/100 (DakoCytomation)	Negative	Negative
Calretinin	1/100 (DakoCytomation)	Negative	Negative
HBME-1	1/100 (DakoCytomation)	Negative	Negative
Beta-catenin	1/200 (Novocastra)	Negative	Negative
